# Baroreflex Activation Therapy in a Patient With Transthyretin Amyloid Cardiomyopathy

**DOI:** 10.1016/j.jaccas.2026.107148

**Published:** 2026-02-26

**Authors:** Naomi R. Khanna, Alexa DenDulk, Gourg Atteya, Sapan N. Talati

**Affiliations:** a Department of Biomedical Engineering, Columbia University, New York, New York, USA; b Advanced Cardiology LLC, Hackettstown, New Jersey, USA; c Morristown Medical Center, Morristown, New Jersey, USA

**Keywords:** cardiomyopathy, chronic heart failure, reduced ejection fraction, shortness of breath

## Abstract

**Background:**

Transthyretin amyloid cardiomyopathy (TTR-CM) is a form of nonischemic cardiomyopathy associated with progressive heart failure, persistent symptoms, and reduced quality of life despite advances in medical therapy.

**Case Summary:**

We present a man with TTR-CM and heart failure with reduced ejection fraction who remained severely symptomatic despite guideline-directed medical therapy, cardiac resynchronization therapy, and atrial fibrillation ablation. Baroreflex activation therapy (BAT) using the Barostim device was implanted as adjunctive therapy, resulting in marked symptomatic improvement and significant gains in quality-of-life metrics.

**Discussion:**

BAT is an established therapy for selected patients with refractory heart failure. Its role in TTR-CM–related cardiomyopathy has not been well described and may offer symptomatic benefit when conventional therapies are insufficient.

**Take-Home Message:**

BAT may be a useful adjunctive therapy to improve symptoms and quality of life in selected patients with TTR-CM and refractory heart failure.

## Graphical Abstract

**Figure:**
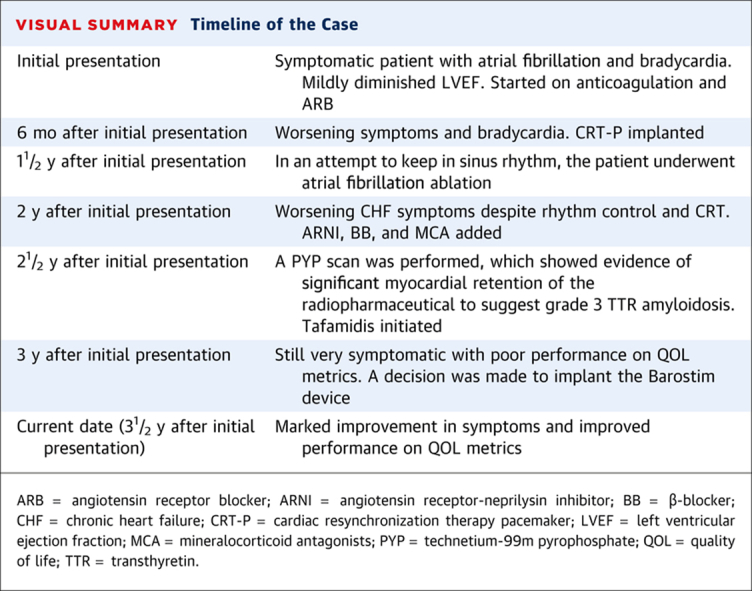

## Case Presentation

The patient is an 86-year-old man seen initially 3.5 years ago with symptoms of shortness of breath and episodes of lightheadedness.

**Take-Home Messages**

• This case report aims to awaken clinicians' recognition of the potential of recommending BAT devices as a means of controlling symptoms in nonischemic cardiomyopathy secondary to TTR-CM.
• Clinicians may want to consider BAT as a potential means of managing worsening symptoms and improving quality of life in TTR-CM patients with a low left ventricular ejection fraction.

**Figure 1:**
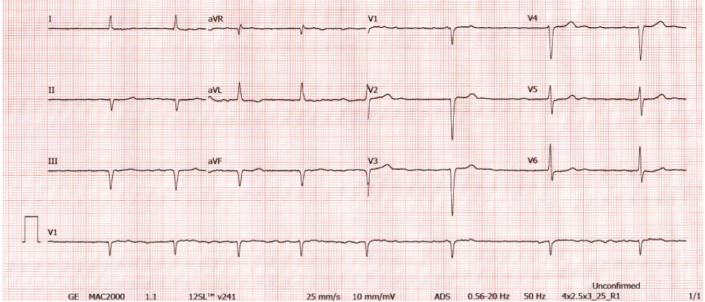
Electrocardiogram Showing Atrial Fibrillation With Slow Ventricular Response and Inferior and Septal Q Waves

Physical examination was notable for an irregular heartbeat with a pulse rate of 50 beats/min. An electrocardiogram confirmed atrial fibrillation with a slow ventricular response and inferior and septal Q waves (Figure 1).

The past medical history was significant only for hyperlipidemia.

The initial differential diagnosis was symptomatic atrial fibrillation, with likely bradycardia and underlying coronary artery disease.

**Figure 2:**
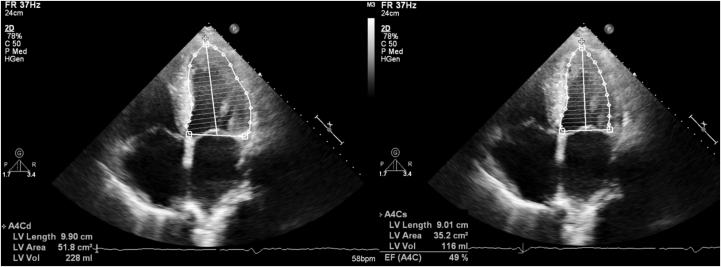
Echocardiogram (Diastolic Image on the Left, Systolic Image on the Right) Demonstrating a Left Ventricular Ejection Fraction of Approximately 45%

His initial blood work results were normal. The echocardiogram was significant for left ventricular hypertrophy and grade II diastolic dysfunction. The left ventricular ejection fraction (LVEF) was 45% (Figure 2). There were no valvular abnormalities that would have warranted intervention. A myocardial perfusion scan suggested inferior ischemia, but subsequent coronary angiography revealed nonobstructive disease.

The patient was initially started on anticoagulation and an angiotensin receptor blocker (valsartan).

**Figure 3:**
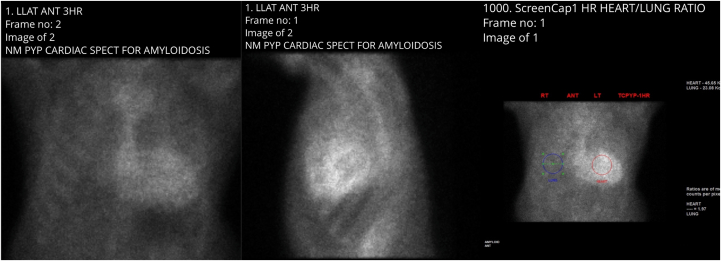
PYP Scan From left to right: 180-minute anterior view, 180-minute left lateral view, and heart-to-lung (H/CL) ratio. Diffuse myocardial retention of tracer is noted on the anterior view. H/CL ratio is 1.92 (normal ≤1.5). Single-photon emission computed tomography (SPECT) demonstrates marked diffuse uptake within the myocardium. PYP = technetium-99m pyrophosphate.

**Figure 4:**
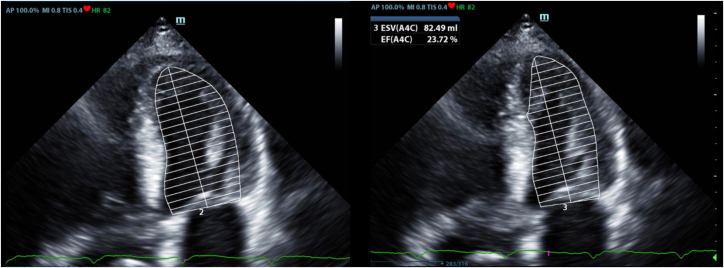
Echocardiogram (Diastolic Image on the Left, and Systolic Image on the Right) Showing a Left Ventricular Ejection Fraction of Approximately 25%

His dyspnea, however, continued to worsen, and his subsequent echocardiogram revealed a drop in his LVEF to 40%. Over the following year, he underwent the placement of a cardiac resynchronization therapy pacemaker (CRT-P). In an attempt to maintain sinus rhythm, he underwent a successful ablation for atrial fibrillation and remained in sinus rhythm. An angiotensin receptor-neprilysin inhibitor (sacubitril) was added to his angiotensin receptor blocker, and he was initiated on a β-blocker (metoprolol), a mineralocorticoid antagonist (spironolactone), and a sodium-glucose cotransporter 2 inhibitor (empagliflozin). Given his progressive symptoms, a technetium-99m pyrophosphate scan was performed, which showed evidence of significant myocardial retention of the radiopharmaceutical to suggest grade 3 transthyretin amyloidosis (Figures 3 and 4). Tafamidis therapy was added to his medical regimen.

**Figure 5:**
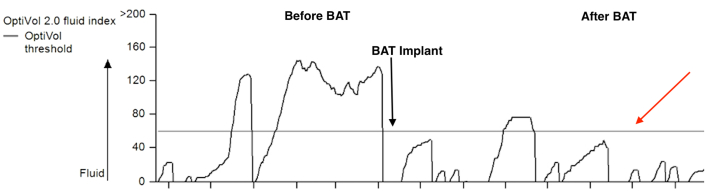
Improvement in OptiVol 2.0 Index after BAT OptiVol 2.0 index measured by the cardiac resynchronization therapy pacemaker demonstrates improvement after baroreflex activation therapy (BAT). The red arrow indicates the threshold for fluid overload.

Despite this aggressive regimen, he continued to decline and had to be admitted twice to the hospital with symptoms of heart failure. In an outpatient visit, his LVEF had dropped to 25% (Figure 5). The 6-minute walk test^1^ was 39.5% of the expected distance (606 feet). His Minnesota Heart Failure Score^2^ was 49. His OptiVol 2 index, as measured by the CRT-P, trended to values far higher than 60, suggesting fluid overload (Table 1, Figure 5). He was deemed to have failed guideline-directed medical therapy (GDMT) and to be a CRT-P nonresponder.

**Table 1:** 6-Minute Walk Test, Minnesota Heart Failure Scores, and OptiVol 2.0 Index Trend Before and After 6 Months Post BAT Implant

Time of Test	The 6-min Walk Test	Minnesota Heart Failure Score	OptiVol 2 Index Trend
Pre-Barostim implant	39.5% of expected distance (606 feet)	49	Above 60
6-mo follow-up	56.73% of expected distance (871 feet)	21	Below 60

The patient received Barostim baroreflex activation therapy (BAT) to address the persistence of symptoms despite GDMT.

Over the following 2 months of receiving Barostim, symptoms improved dramatically. Activity increased to riding a bike again for an hour at a time and walking more than 1 mile at a time.

On his 6-month follow-up visit, his 6-minute walk test had gone up to 56.73% of expected distance (871 feet), his Minnesota Heart Failure Score improved to 21 (Table 1), and his OptiVol 2 index dropped to below threshold levels (Figure 5, Table 1). His weight, blood pressure, and heart rate remained unchanged. The remainder of his medical regimen also remained unchanged, and there were no other interventions that, in our view, would explain the patient's clinical improvement.

## Discussion

Transthyretin amyloid cardiomyopathy (TTR-CM) is a type of nonischemic cardiomyopathy due to the accumulation of transthyretin amyloid protein outside the cell in the myocardial tissue, resulting in cardiac dysfunction.^3^, ^4^, ^5^, ^6^ Initially thought to be very uncommon, with the advent of treatments and diagnostic modalities, increased focus and testing has revealed TTR-CM to be the most frequent cause of diastolic heart failure related to infiltrative heart disease.^3^ The initial symptoms are often subtle and include fatigue, dyspnea, and exercise intolerance. Cardiac arrhythmias, especially atrial fibrillation, are seen commonly. The vague symptomatology has often led to underdiagnosis and requires physicians to have a high index of suspicion.^3^^,^^4^ With the advent of focused disease-modifying agents, there has been a greater interest in early diagnosis. In the history and physical, particular attention should be paid to symptoms of heart failure, atrial fibrillation, and other rhythm disturbances.^3^^,^^4^ The echo shows left ventricular hypertrophy with an echogenic myocardium. The left ventricular hypertrophy is disproportionate to the voltage noted on the electrocardiogram, which often shows low voltage.^3^^,^^4^ Magnetic resonance imaging, and especially technetium-99m pyrophosphate scan, can be useful.^3^^,^^4^^,^^6^ Although the initial presentation is heart failure with preserved ejection fraction, the disease progresses from heart failure with preserved ejection fraction to heart failure with reduced ejection fraction (HFrEF), and as seen in the case, presents with progressive reduction in LVEF and worsening quality-of-life (QOL) metrics.^3^

Treatment includes GDMT for heart failure with diuretics, β-blockers, angiotensin receptor blocker, angiotensin receptor-neprilysin inhibitor, and mineralocorticoid antagonists, though often β-blockers and even angiotensin receptor blocker may be poorly tolerated and not be as beneficial.^3^^,^^4^ More recently, disease-modifying drugs have been developed, namely transthyretin stabilizers^3^, ^4^, ^5^, ^6^ or silencers.^3^^,^^6^ Future trends include transthyretin gene editing and antibody-mediated removal of amyloid.^6^ The use of devices such as implantable cardioverter-defibrillators remains unproven.^3^

Even with standard and newer drugs, the progression of the disease can be variable.^4^ Despite GDMT, there can be progression of disease with patients remaining symptomatic with poor QOL matrices.

Barostim, which is a BAT, is a CVRx Inc implantable neuromodulation therapeutic device that can be used in conjunction with medical therapy in an attempt to control heart failure symptoms.^7^ BAT has leads placed adjacent to the carotid sinus to deliver electrical pulses to the carotid baroreceptors.^7^ Electrical stimulation of the carotid baroreceptors results in activation of the baroreflex system with a subsequent increase in the parasympathetic outflow and inhibition of the sympathetic activity.^7^, ^8^, ^9^

BAT has been presented as a new treatment option for patients with HFrEF who remain symptomatic despite GDMT, with a focus on improving QOL.^7^^,^^9^^,^^10^

Although BAT has previously been studied in patients with HFrEF, its role in TTR-CM has not been well described. In this case, when Barostim was added to the patient's existing treatment plan, the patient demonstrated improved exercise tolerance and a lower Minnesota Heart Failure Score, reflecting enhanced QOL.

## Conclusions

This report presents a case of improved symptomatic outcomes in a cardiac amyloidosis patient with the addition of Barostim, a BAT, to his treatment plan, demonstrating a potential use for such devices in nonischemic cardiomyopathy management where the etiology is TTR-CM.

BAT has been previously reported to be a management tool for HFrEF but has not been specifically studied in the TTR-CM population, where treatment may be challenging because of poor response to standard GDMT for HFrEF. This is especially important as TTR-CM is seen as an increasingly common cause of heart failure, especially in the older population.

## Funding Support and Author Disclosures

The authors have reported that they have no relationships relevant to the contents of this paper to disclose.

**Visual Summary:** Timeline of the Case

Initial presentation	Symptomatic patient with atrial fibrillation and bradycardia. Mildly diminished LVEF. Started on anticoagulation and ARB
6 mo after initial presentation	Worsening symptoms and bradycardia. CRT-P implanted
1½ y after initial presentation	In an attempt to keep in sinus rhythm, the patient underwent atrial fibrillation ablation
2 y after initial presentation	Worsening CHF symptoms despite rhythm control and CRT. ARNI, BB, and MCA added
2½ y after initial presentation	A PYP scan was performed, which showed evidence of significant myocardial retention of the radiopharmaceutical to suggest grade 3 TTR amyloidosis. Tafamidis initiated
3 y after initial presentation	Still very symptomatic with poor performance on QOL metrics. A decision was made to implant the Barostim device
Current date (3½ y after initial presentation)	Marked improvement in symptoms and improved performance on QOL metrics

ARB = angiotensin receptor blocker; ARNI = angiotensin receptor-neprilysin inhibitor; BB = β-blocker; CHF = chronic heart failure; CRT-P = cardiac resynchronization therapy pacemaker; LVEF = left ventricular ejection fraction; MCA = mineralocorticoid antagonists; PYP = technetium-99m pyrophosphate; QOL = quality of life; TTR = transthyretin.
